# 

**Published:** 1908-02-29

**Authors:** 


### CONTENTS.

| | PAGE |
|---|---|
| **The Royal Commission on Whiskey and Potable Spirits** ... | 565 |
| **A Mental Clinic for London** ... | 566 |
| **Annotations—** | |
| The Adulteration of Drugs—Damages Against a Cancer Quack—Medical Inspection of School Children... | 567 |
| **Medical Opinion and Movement** ... | 568 |
| **Hospital Clinics—** | |
| Pneumothorax. By J. E. Squire, C.B., M.D. ... | 571 |
| The Modern Treatment of Ophthalmia Neonatorum. By Rosa Ford, M.B. (Lond.) ... | 573 |
| **Clinical Points—** | |
| Graves' Disease and Acute Rheumatism ... | 575 |
| Bilateral Facial Paralysis ... | 576 |
| **Points in Surgery—** | |
| Some Conditions Which may Simulate Appendicitis ... | 577 |
| **Gynæcology—** | |
| The Case-history of Hæmorrhages in the Early Months of Pregnancy... | 579 |
| **Diseases of Children—** | |
| The Prognosis of Valvular Heart Disease ... | 580 |
| **Therapeutics—** | |
| Secret Remedies and Branded Products—III. ... | 581 |
| **Dermatology** ... | 582 |
| **Hospital Administration—** | |
| Practical Sociology: Ill-health as a Cause of Pauperism ... | 583 |
| On the Arrangements for the Operation Theatre for a Small Hospital ... | 585 |
| News and Coming Events ... | 586 |
| **Nursing Administration—** | |
| Fever Nursing ... | 588 |
| **Editor's Letter-Box—** | |
| "Surgery and the General Practitioner" ... | 589 |
| **The Graduates' Medical Schools** ... | 590 |

